# Looking forward at npj Imaging

**DOI:** 10.1038/s44303-024-00030-6

**Published:** 2024-08-21

**Authors:** Rita Strack

**Affiliations:** Npj imaging, https://www.nature.com/npjimaging/

**Keywords:** Microscopy, Sensors and probes, Optical spectroscopy, Imaging, Bioluminescence imaging, 3-D reconstruction, Diffusion tensor imaging, Endoscopy, Fluorescence imaging, Functional magnetic resonance imaging, Magnetic resonance imaging, Magnetoencephalography, Molecular imaging, Optical imaging, Positron-emission tomography, Time-lapse imaging, Ultrasound, Viral tracing, X-ray tomography, Optics and photonics

Some readers may know me from my long-time role as Senior Editor at *Nature Methods*, where I have handled microscopy, imaging, and probes for nearly a decade. In those years, I have learned about a wealth of tools and technologies, met a wide array of brilliant scientists, and read many cutting-edge research papers across journals that serve the biomedical imaging community. So when I was contacted by the editorial team at *npj Imaging* about serving as an Advisory Editor, I was curious. Where would this journal fit into the research ecosystem, and what could I do to help the journal succeed?

As such, I was surprised and excited when I learned that the goals of npj Imaging are distinct from many other imaging, optics, and biotechnology journals. As Chief Editor Tim Witney described in his first editorial (https://www.nature.com/articles/s44303-024-00015-5), *npj Imaging* sets itself apart by being a journal that publishes important research across disciplines, from optical microscopy to molecular imaging, that spans basic biology and translational research. I applaud this ambitious editorial scope. The imaging community is at an incredible moment in history, where we can image from angstroms to humans, and this has immeasurable potential to improve the human condition. Now more than ever, methods to bridge understanding between the nano-, meso-, organism-, and human-scale are needed to fully understand biology in health and disease.

However, bridging the divide that separates different length scales and imaging specialties can be daunting. The expertise needed to build the next cutting-edge super-resolution microscope is quite different from that required to design an MRI probe for use in the clinic, so much so that the researchers and engineers doing this work may not even share the same scientific vocabulary. They may not feel they have similar goals, may not read the same journals or attend the same meetings, and may not have the bandwidth to keep up with what other technologies are capable of.

In creating *npj Imaging*, I hope we’re building a common ground where researchers across disciplines can meet, engage with each other’s work, exchange ideas, and be inspired to create new instruments and methods. In this way, we can serve an exceptionally broad research community and complement existing journals.

When it comes to imaging across scales, I am excited about the possibilities. I have seen growth happening along two major paths. The first is taking individual modalities and pushing them toward their limits in terms of sensitivity, resolution, and coverage. As an example, in my time as a scientist, I have seen super-resolution microscopy go from capturing very small fields of view in two dimensions to whole volumes of cells and even intact spheroids. The second is multimodal imaging which seeks to combine the best of different approaches for improved discovery. Some examples in smart microscopy combine fast, low-resolution imaging of large volumes with super-resolution imaging of only smaller regions of interest containing interesting biological phenomena. I am particularly excited about methods like photoacoustic imaging, which can bridge length scales and connect studies done with fluorescence microscopy and those done with, for example, super-resolution ultrasound imaging in animals.

The future of imaging across scales will involve both types of advances and ideally will involve an increase in multimodal imaging that encompasses both optical and molecular imaging. I envision the future of this space will also involve advances in computer vision for tasks such as data fusion across imaging modalities, data augmentation, image reconstruction, and cross-modality image generation. Even as I type these words, I know that exciting work is already happening along these lines in both basic research and medicine. I also strongly believe that improved probes, especially multimodal probes, are poised to have an outsized impact on the future of imaging technologies, much like the green fluorescent protein shaped bioimaging today.

In my view, the goal of understanding the structure and functional connectivity of every cell across an entire human (and how this changes in disease) is not a pipe dream. Indeed, we will have this and more as genomic information is integrated with more traditional imaging, as has happened with histology and spatially resolved transcriptomics. But it will take serious cross-disciplinary efforts and technological advances to get there. I believe that the next generation of scientists will have a broad understanding of how different technologies can be used to tackle their questions. I hope they make use of cutting-edge microscopy core facilities, medical imaging facilities, and even synchrotrons to view their specimens in diverse ways. I’ve observed that it’s often in using technologies for discovery that we learn what tools are missing. I am excited to see how researchers will explore those gaps and build new tools to address outstanding questions in biology and improve personalized medicine.

I believe *npj Imaging* can stand at the forefront of technological advances that both impact individual modalities and improve imaging across scales. I hope we can gain a diverse readership that comes to the journal for the papers in their domain which stays for the high-quality content from other disciplines. I think we can make this worth the reader’s while, as each paper in the journal is expected to have a strong biomedical application showcasing the benefits of technological advances. In this way, it is not purely a technology journal, but a true biomedical research journal.

So what can I do to help the journal succeed? So far I have served in an advisory capacity, helping build out the editorial board, guiding papers to the journal, and occasionally sharing tips based on my editorial experience. I hope that by writing this piece I’ve helped readers understand why I am so invested in this new cross-disciplinary journal. I invite you to explore the already-excellent content and get a sense of the heart of *npj Imaging*. I encourage those reading this to subscribe to the electronic table of contents and keep tabs on the journal as it grows. Finally, I hope readers submit their papers to this highly visible young journal. Both research papers and Reviews, Perspectives, and opinion pieces will shape the character of the journal and help it develop to its full potential, and we warmly welcome submissions. If you have questions, please reach out to me or my colleagues on the editorial board.

